# First-Principles Studies on the Structural Stability of Spinel ZnCo_2_O_4_ as an Electrode Material for Lithium-ion Batteries

**DOI:** 10.1038/srep36717

**Published:** 2016-11-09

**Authors:** Wei-Wei Liu, M. T. Jin, W. M. Shi, J. G. Deng, Woon-Ming Lau, Y. N. Zhang

**Affiliations:** 1 Beijing Computational Science Research Center, Beijing 100193, China; 2 Chengdu Green Energy and Green Manufacturing Technology R&D Center, Chengdu, Sichuan, 610207, China; 3 Sichuan New Material Research Center, Mianyang, 621000, Sichuan, China; 4 Institute of Chemical Materials, China Academy of Engineering Physics, Mianyang, 621900, Sichuan, China

## Abstract

Systematic first principles calculations were performed for ZnCo_2_O_4_ to clarify its structural and electronic properties, and particularly the structural stability as an electrode material for lithium-ion batteries. For samples with low Li concentration, e.g., Li_*n*_ZnCo_2_O_4_ with *n* < 1, Li atoms take the center of oxygen octahedra and may diffuse rapidly. Structure distortions and volume expansions can be observed in Li_*n*_ZnCo_2_O_4_ with *n* > 1 and amorphous structures eventually prevail. The AIMD simulations for Li_9_ZnCo_2_O_4_ suggest the formation of Li_2_O, Co_3_O_4_ and LiZn local compounds or alloys. In particular, the formation of Zn-Co aggregations and the losing of ZnO pairs are identified as the possible reasons that are responsible to the Li capacity fading in ZnCo_2_O_4_ anodes.

Lithium-ion batteries (LIBs) are now ubiquitous in portable electronics due to their high energy density, low weight and small volume. Intercalation of LIBs to more advanced battery systems has also been used in a verity of devices including electric vehicles^1^,^2^,^3^,^4^. Researchers are continually exploring new electrode materials to further enhance the Li capacity and safety of LIBs, and to reduce the cost. To this end, deep understanding of electrochemical processes during charge-discharge (delithiation-lithiation) cycle of LIBs is essential^5^,^6^. Transition metal oxides (TMOs), such as Fe_3_O_4_, CuO, NiO, and Co_3_O_4_, have been widely used as high-capacity anode materials for LIBs^7^,^8^,^9^,^10^,^11^. Among these, the cubic spinel Co_3_O_4_ has a high capacity of ~900 mAhg^−1^, with 100% capacity retention for up to 25 cycles^12^,^13^. Recently, extensive efforts have been made towards replacing Co in Co_3_O_4_ partially by eco-friendly and cheaper alternative metals, such as Ni^14^ and Zn^15^.

ZnCo_2_O_4_ (ZCO) spinel is a typical *p*-type transparent conducting oxide that combines high optical transparency and high electrical conductivity. It is promising for broad applications in solar cell, smart window and liquid crystal display^16^,^17^. With its high reversible Li capacity, long cycling life and environmental friendliness, ZCO is also an attractive material for the use in LIBs and supercapacitors^18^,^19^,^20^,^21^,^22^,^23^. Previous ex-site TEM^24^ and ex-site X-ray diffraction (XRD) studies^25^ have established that the electrochemical reactions of ZCO with Li during a charge-discharge cycle include several steps:
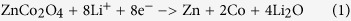

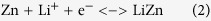

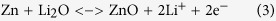

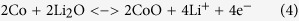

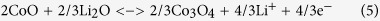


One may therefore expect a total capacity corresponding to ~8.33 mol of recyclable Li per mole of ZCO, with the formation of metal oxides and decomposition of Li_2_O^18^. However, the real capacity of ZCO-based LIBs decays quickly upon Li charge-discharge cycles. A few dozen studies have explored the possible causes of capacity degradation, and have identified several possible factors such as the large volume changes and subsequent mechanical instabilities in electrodes^26^,^27^,^28^,^29^, the formation of a solid electrolyte interphase (SEI)^30^, and the reduction of metal oxide to metal with the formation of Li_2_O^23^. No consensus has been reached yet and further fundamental studies are desired for the development of superb anode materials in LIBs. At the present stage, theoretical studies have been focused on the structure and electronic properties of ZCO^31^,^32^,^33^, and very few touched on structural transformation and stability during the electrochemical reaction process. According the reactions listed above, the charging and discharging processes involve local chemical reaction and structure destruction, a case that differs significantly from the classical lithiation/delithiation processes in the layered or olivine electrode materials. The appropriate descriptions on the entire electrochemical process of Li atoms in spinels are still challenging tasks for theoretical studies.

In this work, we study the structural and electronic properties of ZnCo_2_O_4_ by using first-principles calculations. The structural stability, Li diffusion, and the key electrochemical reaction steps of Li_*n*_ZnCo_2_O_4_ after lithium insertion are simulated and analyzed. We find that, the electronic characters of ZCO ground state, a nonmagnetic cubic spinel structure, are mainly dominated by alignment and hybridization between the Co-3*d* and O-2p orbitals. The structure of Li_*n*_ZnCo_2_O_4_ is stable for a small lithium capacity, e.g., *n* ≤ 1, and the energy barrier for Li atom diffusing from the center of one oxygen octahedron to its adjacent octahedron is about 0.4 eV. For cases with *n* > 1, the structure becomes locally disordered with a large volume expansion as large as 180%. The pair correlation functions of the final state of Li_9_ZnCo_2_O_4_ show the formation of ZnCo network, instead of ZnO, during the electrochemical reactions of ZCO with Li, which leads to a reduction of the reversible lithium capacity.

## Results and Discussions

### Structure and electronic properties of ZCO

**Figure 1:**
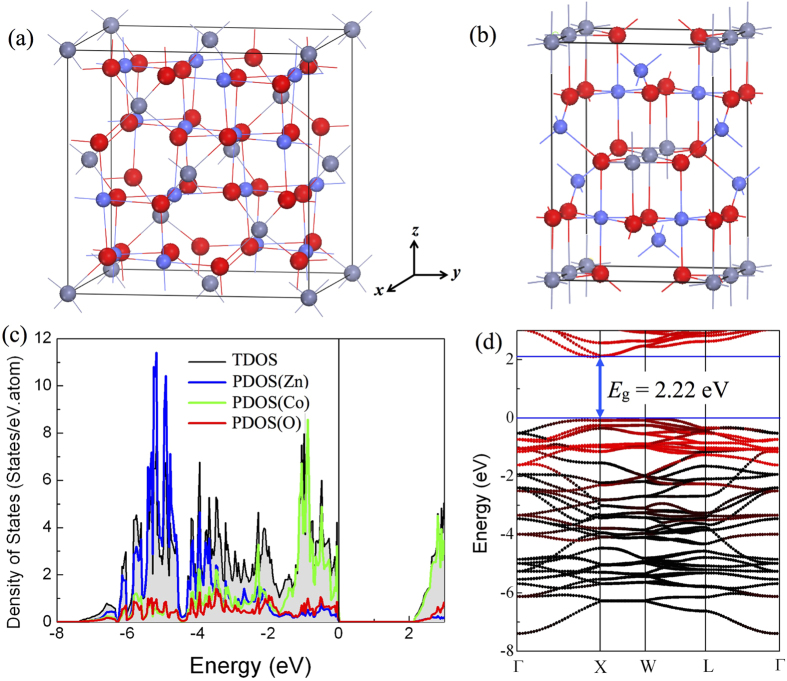
(**a**) The cubic spinel and (**b**) tetragonal spinel structures of ZnCo_2_O_4_. Grey, blue and red balls represent Zn, Co and O atoms, respectively. (**c**) The total (the black line with gray shadow) and projected density of states of Zn, Co and O atoms. (**d**) Band structure, with a red color indicating the contributions from the Co atom. The horizontal blue lines indicate the energy positions of the VBM and CBM.

Zinc oxide spinels may have different configurations, depending on the positions of Zn and Co atoms. If the tetrahedron sites (T_d_, tetrahedrally coordinated) in Co_3_O_4_ are only occupied by Zn^2+^, its structure is a cubic spinel, as shown in Fig. 1(a). When Zn^3+^ stay on the octahedral sites (O_h_, octahedrally coordinated), and Co^3+^ and Co^2+^ occupy the octahedral and tetrahedron center, respectively, the structural deformation occurs to form a tetragonal spinel structure, as shown in Fig. 1(b). The phase transition between these two structures happens under certain temperature or pressure^34^,^35^. With the GGA+U functional, our energy calculations of the cubic and tetragonal spinels show that ZCO is a nonmagnetic (NFM) cubic spinel structure. The calculated lattice constant, 8.164 Å, and band gap, 2.22 eV, are in good agreement with the corresponding experimental values of 8.0946(2) Å (JCPDS card no. 23–1390) and 2.26 eV, respectively^36^. Note that the regular GGA method gives a similar lattice constant but a very small band gap of only 0.60 eV. The ground magnetic state of the tetragonal spinel is ferromagnetic and its energy is higher than that of the cubic spinel by about 2.30 eV, indicating that the phase transition between the two phases of ZCO is almost impossible under normal experimental conditions.

The calculated total and projected density of states in Fig. 1(c) show that the O-2*p* orbitals have a weak hybridization with Co-3*d* states just below the Fermi level. Both Co-3*d* and O-2*p* orbitals determine the valance band maximum (VBM) of ZCO, whereas the conduction band minimum (CBM) is mainly dominated by the Co-3*d* states. The Zn atom is relatively inert in determining the band edges of ZCO, but it bridges the interactions between tetrahedrally coordinated Co-3*d* and O-2*p* electrons. These features can be clearly seen from the band structure in Fig. 1(d), where the red color indicates the contributions from the Co atom. We see that the ZCO spinel has an indirect band gap: the VBM is near the W point along the W-L direction and the CBM is located near the X point along the Γ-X direction. The VBM of ZCO is characterized by a very flat dispersion, which results in heavy holes with large effective masses and may lead to poor *p*-type conductivity.

### Electrochemical properties of Li_n_ZnCo_2_O_4_

**Figure 2:**
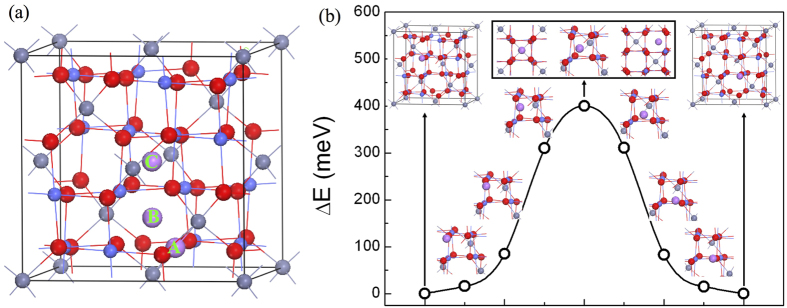
(**a**) Three possible models of inserting one Li atom in the supercell denoted as A, B and C. (**b**) The energy barrier of one Li atom diffusing in a 2 × 2 × 2 ZCO supercell. The insets show the local structures at each step. The purple ball is the Li atom.

We may understand the structural stability and electrochemical properties of ZnCo_2_O_4_ upon lithiation process starting from one Li atom insertion in ZCO, i.e., Li_0.125_ZnCo_2_O_4_. Here we constructed three possible sites for the Li atom in the supercell shown in Fig. 2(a), denoted as A, B and C. For site A, the Li atom locates in the octahedral center surrounding by six oxygen atoms; Li stays in the lattice channel of the structure in site B, and it is in the “cage” consisting of four Co atoms and four O atoms in site C. All the three configurations are stable after structure relaxation, and the site A has the lowest energy. The energy difference between the site A and site C is as high as 2.67 eV. So the possibility of having Li on site C is negligible and the diffusion pathway of Li atom in ZCO should be along A −> B −> A. The relative energy and local structure for each step calculated by the nudged elastic band (NEB) method^37^,^38^ are shown in Fig. 2(b). Here we use a GGA method to avoid the energy disturbance caused by charge transition on the energy barrier^39^,^40^,^41^. We can see that the Li atom leaves the center of the oxygen octahedron for the transition state, i.e. the site B, and then moves to the adjacent octahedron. The calculated energy barrier of Li diffusion is about 0.4 eV as shown in Fig. 2(b), which should be easy to overcome under the normal experimental conditions.

**Figure 3:**
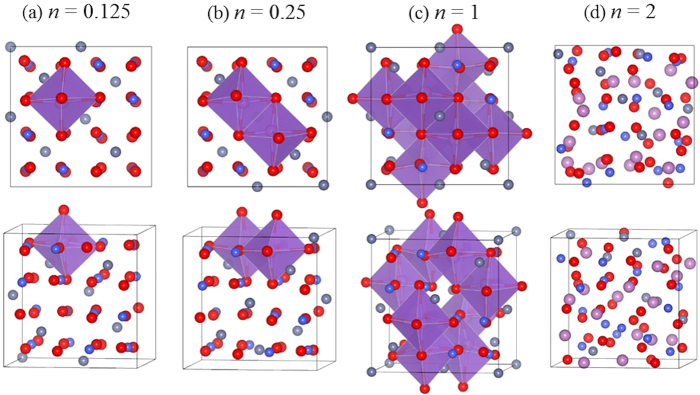
The structures of LinZnCo_2_O_4_ with n = (**a**) 0.125, (**b**) 0.25, (**c**) 1 and (**d**) 2. The grey, blue, red and purple balls represent Zn, Co, O and Li atoms, respectively, and Li atoms are in the center of the blue octahedrons in (**a**–**c**).

We then insert more Li atoms in the supercell to observe the structural stability of Li_n_ZnCo_2_O_4_ and the lithium capacity of ZCO. For each *n*, we searched different configurations, and those with the lowest energies are shown in Fig. 3(a–c) for *n* = 0.125, 0.25 and 1. Interestingly, we found that the second Li atom tends to stay in the center of the nearest neighbor oxygen octahedron, which is consistent with the diffusion channel of one Li atom we discussed above. For *n* ≤ 1, the structure of Li_n_ZnCo_2_O_4_ is stable until all octahedral “cages” in ZCO have been fully occupied, and the volume of LiZnCo_2_O_4_ expands by ~10% compared with that of ZnCo_2_O_4_. Note that the positions of Zn atom are not in the ideal lattice site already. So the nearest neighbor distance between Zn and Zn atoms shrinks from 3.3 Å to 2.4 Å. As *n* > 1, such as for *n* = 2 in Fig. 3(d), the atoms in Li_n_ZnCo_2_O_4_ are clearly not in the lattice sites of cubic spinel and the crystalline structure starts to distort.

**Figure 4:**
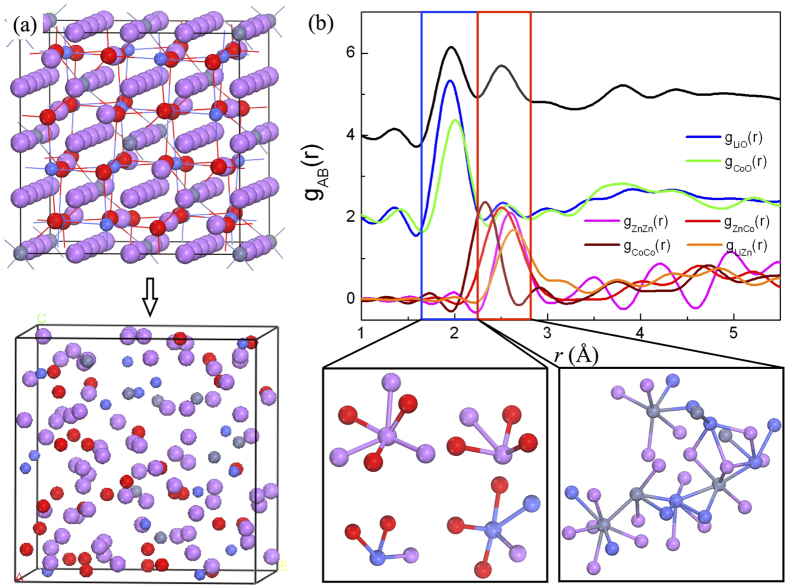
(**a**) The initial (upper panel) and final (lower panel) states of Li_9_ZnCo_2_O_4_ by using AIMD method. (**b**) The total and partial pair correlation functions of the Li_9_ZnCo2O_4_ final state. The left and right insets show the local atomic pairs with a band-length range of 1.65–2.25 Å (blue rectangle) and 2.25–2.80 Å (red rectangle), respectively.

According to Eqs (1, 2, 3, 4, 5), one can expect a ratio of 9 mol of Li in a mole of ZCO. We thus examined Li_9_ZnCo_2_O_4_ for the high concentration case through AIMD studies. The initial and final structures are depicted in the upper and lower panels in Fig. 4(a). As expected, the structure of Li_9_ZnCo_2_O_4_ is disordered and its volume expands by ~180% compared with that of clean ZnCo_2_O_4_. The local chemical order in Li_n_ZnCo_2_O_4_ alloys can be directly characterized by the partial pair correlation functions (PCF, *g*_AB_), which is defined as the number of B-type atoms in the spherical shell ranging from *r* to (*r* + d*r*) around an *A*-type atom. Quantitatively, it is calculated by:
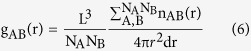
where *L* is the lattice length of the 128-atom cubic unit cell, *N*_*A*_ and *N*_*B*_ are the numbers of *A* and *B* atoms, respectively, in the unit cell, and *n*_*AB*_ is the average number of *A*-*B* pairs with a saparation *r*.

The total pair correlation function of Li_9_ZnCo_2_O_4_, *g*_tot_(*r*), shows broad peaks in Fig. 4(b), indicating the amorphous feature of the final structure. The first peak of *g*_tot_(*r*) at ~2.0 Å primarily results from Li-O and Co-O pairs, indicating the formation of Li_2_O and Co_3_O_4_ local structures Some Li-O and Co-O pairs have a bond length range of 1.65–2.25 Å, as highlighted in the blue rectangle in Fig. 4(b). Another peak of *g*_tot_(*r*) at ~2.5 Å represents Zn-Zn, Zn-Co, Co-Co and Li-Zn pairs. Interestingly, we searched Zn- and Co- pairs within a bond length range of 2.25–2.80 Å in the red rectangle, and clearly saw the formation of Zn-Co network with some Li atoms connecting with Zn atoms, as depicted in the right inset in Fig. 4(b). On the contrary, we can rarely find the Zn-O pairs, indicating that more Zn atoms interact with Co atoms instead of O. Y. Sharma *et al.* have confirmed that Zn- and Co- nano-particles contribute to the stability and high lithium capacities of ZCO through both alloy formation and displacement reaction, namely LiZn ↔ Zn ↔ ZnO and Co ↔ CoO ↔ Co_3_O_4_^18^. The lack of ZnO breaks the mutual beneficial matrices and makes the reversible Li capacity of ZCO to be smaller than 6.33 mol in real experiments according to the Eq. (3).

**Figure 5:**
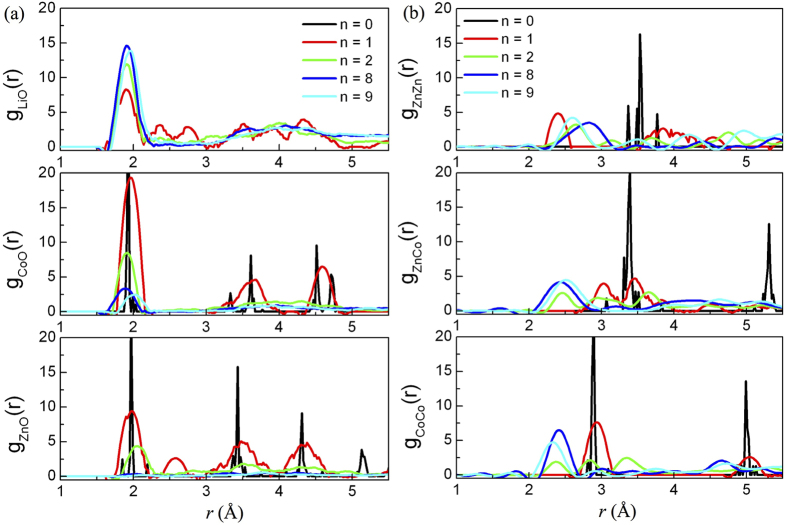
The partial pair correlation functions of Li_n_ZnCo_2_O_4_ as a function of n.

We also studied the changes of atomic pairs as a dependence of *n* value in Li_n_ZnCo_2_O_4_, as shown in Fig. 5, to observe the structure evolution upon lithiation process. With the addition of Li atoms, *g*_LiO_(r) increases but *g*_ZnO_(r) and *g*_CoO_(r) decrease quickly. In particular, *g*_ZnO_(r) is almost zero for *n* = 8 and 9, as shown in the lower panel in Fig. 5(a). The positions of the first peaks of *g*_LiO_(r), *g*_CoO_(r) and *g*_ZnO_(r) are almost unchanged compared with the ideal ZCO, which illustrates the stability of these metal oxides. However for *g*_ZnZn_(r), *g*_ZnCo_(r), and *g*_CoCo_(r), the positions of their first peaks shown in Fig. 5(b) obviously change with the insertion of Li atoms and there are broad peaks at around 2.45 Å. In contrast with the decrease of *g*_ZnO_(r), the number of Zn-Zn, Zn-Co and Co-Co pairs increases with the increasing Li composition. Therefore, one may expect Zn-Co aggregation in real samples after several rounds of lithiation/delithiation cycles, which might be responsible to the decrease of Li capacity. A stable Li capatity could occur in nanophase or porous ZnCo_2_O_4_ materials due to the inhibition of Zn-Co network and the flexibility of nanograins. More experiments are expected for the verification of our theoretical results.

In summary we performed systematic density functional studies on the structure, magnetic, and electronic properties of ZnCo_2_O_4_, as well as the lithium diffusion and structural stability upon lithiation process as an anode material of LIBs. It was shown that ZCO is a nonmagnetic cubic spinel structure with an indirect band gap of 2.22 eV by using GGA+U functional. Lithium atom in ZCO prefers to occupy the center of oxygen octahedron and the energy barrier of one Li atom diffusing to the adjacent octahedral center is about 0.4 eV. While the structure of Li_n_ZnCo_2_O_4_ is stable for small lithium capacity, it becomes locally disordered as Li: Zn >1 mol: 1 mol with a volume expansion of >180%. We further found the formation of Zn-Co network instead of ZnO alloy in the final structures of Li_9_ZnCo_2_O_4_ through AIMD calculations. So the structure destruction occurs in the lithiation process that makes the reversible lithium capacity fading during cycles. Our extensive calculations provide instructive information for understandings of experimental results and also give useful insights for the design and optimization of high rate electrode materials.

## Method

Spin-polarized density functional calculations were performed by using the Vienna *Ab initio* Simulation Package (VASP)^42^ along with the projector augmented wave (PAW) method^43^. The Perdew-Burke-Ernzerhof (PBE) formulation of the generalized-gradient approximation (GGA)^44^ was adopted to describe the exchange-correlation interaction among electrons, and a Hubbard *U* of 4 eV (GGA+U) was added for Co 3*d* orbitals^45^,^46^. Throughout this work, we used an energy cutoff of 500 eV for the plane wave expansion. The convergence of our results against the *k*-points sampling in the Brillouin zone was carefully examined for all cases, for example, an 11 × 11 × 11 Monkhorst-Pock *k*-point set for a primitive cell. The crystal constant and positions of the ions were fully relaxed until the final force on each atom is smaller than 0.01 eV/Å. Gaussian smearing method with a smearing width of 0.05 eV was used to accelerate the convergence. We used a 2 × 2 × 2 supercell (128 atoms) to study the structural stability of Li insertion in ZCO, as well as the diffusion channels and energy barrier of Li atoms.

We did *Ab initio* molecular dynamics (AIMD) calculations for the determination of the final structures and reaction products upon lithiation processes. The atomic spacing and positions were fully optimized in a cubic supercell and the annealing process was performed at 300 K for 5 ps in a canonical (NVT) ensemble with a time step of 3 fs. While only the Γ-point was used to sample the Brillouin-zone during the annealing process, 3 × 3 × 3 Monkhorst-Pack *k*-points were used for the geometry relaxation and electronic structure determination after the AIMD simulations.

## Additional Information

**How to cite this article**: Liu, W. W. *et al.* *First-Principles* Studies on the Structural Stability of Spinel ZnCo_2_O_4_ as an Electrode Material for Lithium-ion Batteries. *Sci. Rep.* **6**, 36717; doi: 10.1038/srep36717 (2016).

**Publisher’s note**: Springer Nature remains neutral with regard to jurisdictional claims in published maps and institutional affiliations.
